# *SIRT6* Minor Allele Genotype Is Associated with >5-Year Decrease in Lifespan in an Aged Cohort

**DOI:** 10.1371/journal.pone.0115616

**Published:** 2014-12-26

**Authors:** Mindi J. TenNapel, Charles F. Lynch, Trudy L. Burns, Robert Wallace, Brian J. Smith, Anna Button, Frederick E. Domann

**Affiliations:** 1 Department of Epidemiology, College of Public Health, The University of Iowa, 01620 PFPW, 200 Hawkins Drive, Iowa City, Iowa, United States of America; 2 Free Radical & Radiation Biology Program, Department of Radiation Oncology, The University of Iowa, Iowa City, Iowa, United States of America; CSIR-Central Drug Research Institute, India

## Abstract

Aging is a natural process involving complex interplay between environment, metabolism, and genes. Sirtuin genes and their downstream targets have been associated with lifespan in numerous organisms from nematodes to humans. Several target proteins of the sirtuin genes are key sensors and/or effectors of oxidative stress pathways including *FOXO3*, *SOD3*, and *AKT1*. To examine the relationship between single nucleotide polymorphisms (SNP) at candidate genes in these pathways and human lifespan, we performed a molecular epidemiologic study of an elderly cohort (≥65 years old.). Using age at death as a continuous outcome variable and assuming a co-dominant genetic model within the framework of multi-variable linear regression analysis, the genotype-specific adjusted mean age at death was estimated for individual SNP genotypes while controlling for age-related risk factors including smoking, body mass index, alcohol consumption and co-morbidity. Significant associations were detected between human lifespan and SNPs in genes *SIRT3*, *SIRT5*, *SIRT6*, *FOXO3* and *SOD3*. Individuals with either the CC or CT genotype at rs107251 within *SIRT6* displayed >5-year mean survival advantages compared to the TT genotype (5.5 and 5.9 years, respectively; *q*-value  = 0.012). Other SNPs revealed genotype-specific mean survival advantages ranging from 0.5 to 1.6 years. Gender also modified the effect of SNPs in *SIRT3*, *SIRT5* and *AKT1* on lifespan. Our novel findings highlight the impact of sirtuins and sirtuin-related genotypes on lifespan, the importance of evaluating gender and the advantage of using age as a continuous variable in analyses to report mean age at death.

## Introduction

Approximately 35% of individual differences in human longevity are estimated to be heritable [1]. The science of aging has made tremendous strides since the discovery in the 1980s that specific gene mutations in yeast, worms and flies can extend lifespan [2]. However, extrapolation of these findings to the complex human body remains a major challenge due to differences in physiology and aging-related diseases. Nevertheless, animal models have provided key insights to candidate genes and pathways that provide a focus for investigations in humans. Included among these are the family of nicotinamide adenine dinucleotide (NAD) dependent protein deacetylases, including the Sirtuins [3]. Initial experiments within *Caenorhabditis elegans* indicated a duplication containing *sir-2.1*, the gene most homologous to yeast *SIR2*, increased lifespan by 50% [4]. However, subsequent studies suggested a second mutation within the *dyf* gene was responsible for the majority of the increase in lifespan [5]. When controlling for the second mutation, the effect of *sir-2.1* on lifespan was reduced from a 50% increase to a 10–15% increase.

Sirtuins have also been linked to oxidative stress through genetic manipulation of Sir2 in eukaryotic model organisms [6]. Calorie restriction has been suggested to slow aging by reducing levels of reactive oxygen species. Lifespan extension observed during calorie restriction in yeast was not seen in strains mutant for Sir2, suggesting increased lifespan induced by calorie restriction requires the activation of the Sir2 protein [7].

Mammals have seven sirtuin genes (*SIRT1-7*) comprising the sirtuin family of proteins (SIRT1-7). The proteins vary in tissue specificity, subcellular localization, enzymatic activity and targets [8]. SIRT1, SIRT6 and SIRT7 are found in the nucleus, SIRT2 is found in the cytoplasm, and SIRT3, SIRT4 and SIRT5 are primarily found in the mitochondria. *SIRT1*, *2* and *3* have been shown to control the oxidative stress response by regulating the FOXO family of Forkhead transcription factors and elevating downstream targets such as manganese superoxide dismutase (MnSOD or SOD2) [9]–[11]. *SIRT1* has also been demonstrated to mediate the p53 regulatory pathway, decreasing the sensitivity of cells to DNA damage and oxidative stress [12]. Although not proven, a causal role for oxidative stress in aging remains one of the most widely accepted aging theories [6].

In addition to being downstream targets of sirtuins, variants in *FOXO1*, *FOXO3* and *p53* have shown independent associations with human lifespan [13]–[15]. *CAMKIV* has been shown to be associated with lifespan through activation of survival proteins AKT1, SIRT1 and FOXO3A [16].

The variability in individual lifespan, not attributed to genetic variation, and changes in life expectancy over the past century have largely been attributed to environmental factors including smoking, body mass index and alcohol consumption [17]. The importance of investigating the impact of genetic variation on lifespan while controlling for these environmental factors and disease occurrence is pivotal. The aim of this study was to evaluate the association of sirtuin single nucleotide polymorphisms (SNPs) with age at death while controlling for aging-related risk factors, including gender, body mass index, exercise, comorbidities, smoking and alcohol consumption. Additional gene variants located within candidate oxidative stress genes and genes shown to interact with the sirtuin family of genes were also evaluated.

## Methods

### Study Population

The Established Population for Epidemiologic Studies of the Elderly (EPESE) project was initiated by the intramural Epidemiology, Demography and Biometry Program of the National Institute on Aging in 1980. This study has been described in detail [18]. Four populations were included in the project; the Iowa population was the focus of this analysis. The Iowa component of EPESE “Iowa 65+ Rural Health Study” (65+RHS) targeted residents living in two Iowa counties, Washington and Iowa, aged 65 and older as of September 1, 1981. Approximately 4600 persons who were 65 years and older were contacted and 80% agreed to participate, resulting in an initial cohort of 3673. Upon obtaining consent, the 65+RHS baseline survey was completed in the participant's home during 1982–83, with seven follow-up interviews through 1992 [18]. The baseline, third and sixth follow-up surveys were conducted in the participant's home by a trained interviewer [19]. At the sixth follow-up interview, during 1987–89, 1940 of 2739 participants agreed to provide a blood sample (71% of those re-interviewed). Blood was drawn and then frozen at −70°C until thawed for assay. Only 1260 of the 1940 blood specimens were available for this study. Participants whose blood was not analyzed included those who chose not to donate blood (n = 799), missing samples (n = 680), and samples that did not have a genotype call rate ≥90% (n = 68). This project was reviewed and approved by the University of Iowa IRB. Participant information was anonymized prior to analysis.

### Ascertainment of Death

Death dates for nearly all participants were provided through the National Death Index in a linkage completed in 2009 [20]. No death date was identified for approximately 120 of the cohort members at the time of the linkage. For these 120, a linkage with the State Health Registry of Iowa (SHRI) death certificate database in April, 2012 and a search of the Social Security Death Index in April, 2013 were used in efforts to obtain date of death. Death dates for all but 17 participants who donated blood were found. For these participants date of last known contact was used for age at death.

### Aging-Related Risk Factor Data Collection

Aging-related risk factors were not of direct interest in the analysis reported herein and were only included to control for confounding. Information collected in the baseline survey on demographic and lifestyle characteristics in the 65+RHS included smoking, alcohol consumption and physical activity. Several participants either refused or did not supply these data. To include all participants in the analysis an unknown category was created for missing data. To ensure the unknown category was not directly impacting results, sensitivity analyses excluding unknowns were conducted to ensure the general magnitude and direction of association was similar.

Participants were categorized as ever vs. never smokers. Ever smokers included cigarette, pipe, cigar and occasional smokers. These were grouped since the subgroups were too small to have sufficient power for an association analysis. For alcohol consumption, participants were classified as never, former, current low or current high. Collapsed categorical variables were not considered for alcohol consumption due to abstinence and high consumption having an elevated mortality risk relative to moderate consumption. An average of 1 drink per day for females and 2 drinks per day for males was used to distinguish between current low and current high alcohol consumption [21]. Self-reported height and weight were used to calculate body mass index (BMI) and categorized according to the World Health Organization's definitions [22]. Participants were asked about exercise and daily tasks to assess physical activity. Responses were used to classify participants as inactive, moderately active or highly active based on two previously published EPESE reports [19], [23]. Previous EPESE analyses utilized the health history portion of the baseline survey to create a comorbidity score [24]. Participants were asked if a doctor had ever told them they had the following conditions: myocardial infarction, stroke, diabetes, cancer or hypertension and were categorized as having 0, 1 or>1 comorbidities for analysis.

### SNP Selection and Genotyping

The International HapMap Project was utilized to download SNP genotype data based on Utah samples. Of the genotyped populations available through HapMap, the Northern and Western Europe (CEU) population was most similar in ancestral origin to the 65+RHS. DNA from select CEU individuals also served as controls during genotyping.

SNP genotype data were downloaded for the following genes: *SIRT1, SIRT2, SIRT3, SIRT4, SIRT5, SIRT6, SIRT7, AKT1, CAMK4, FOXO1, FOXO3, GPX1, SOD2, SOD3* and *TP53*. This information was uploaded into Haploview 4.2 using a specified minor allele frequency of 0.05. For *SIRT1, 2, 3* and *6* the tagger configuration was forced to include all SNPs reported in the literature and used to determine which additional SNPs were needed for entire gene coverage. For *SIRT4, 5* and *7*, as well as the other candidate genes, a literature search was conducted to identify SNPs associated with aging and other diseases. If no literature was found, Haploview was used to select the SNP(s) that tagged for the highest number of additional SNPs.

Entire gene coverage, as determined by Haploview 4.2, was met for *SIRT1* and *SIRT6*. For *SIRT3* no assay for one of the selected SNPs, rs12222188, was available through TaqMan. Even with this SNP excluded, 97% of the genetic information was captured by the other 12 SNPs that were identified.

Genotyping was performed on 1260 individuals at 48 known SNPs using Fluidigm 192.24 Dynamic Array integrated fluid circuits (Fluidigm Incorporated, San Francisco, CA). Samples and Taqman SNP genotyping assays (Life Technologies, Grand Island, NY) were loaded into reaction chambers by the IFC Controller RX and a fast PCR protocol was run on the FC1 Cycler. The chip was read using fluorescence detection by the BioMark HD Reader and the Fluidigm analysis software v3.1.2 was used to view and interpret results. All genotype data were uploaded to the database Progeny 8, which detects and flags all genotyping discrepancies. All samples with a call rate of ≤90% were excluded from analyses (n = 68).

### Statistical Analysis

Participants of the Iowa 65+RHS study are representative of an elderly Iowa, Caucasian population [18]. Student's t-tests and Pearson's chi-square tests were used to compare covariate distributions between participants whose blood was analyzed *versus* those without a blood sample. The baseline interview was used to capture covariates for all participants.

Agreement of observed genotype frequencies with expected Hardy-Weinberg equilibrium frequencies was assessed. Linkage disequilibrium (LD) was assessed between all SNP pairs using the correlation coefficient, r^2^. SNP pairs with an r^2^ of ≥0.8 were considered to be in high LD; pairs with an r^2^ of 0.5–0.8 were considered to be in moderate LD. The Kruskal-Wallis test was used to evaluate SNP-covariate associations.

Multivariable linear regression analysis was used to assess SNP-age at death associations while controlling for aging-related factors. A base model was constructed to determine which variables were significantly associated with age at death prior to the inclusion of genotype data. Variables considered were gender, smoking status, alcohol consumption, body mass index (BMI) category, comorbidity count, exercise classification and all two-way interaction effects. Based on the corrected Akaike Information Criterion (AICc) in backward selection, components of the pre-genotype base model included: gender, comorbidity count, smoking, alcohol consumption, BMI and the BMI x smoking interaction effect. Exercise was not significant in the base model and therefore was not included in any subsequent analyses.

To assess the impact of each genetic variant on age at death a co-dominant genetic model was assumed [25] and each SNP in turn was added to the base model. Partial correlation coefficients were calculated to estimate the strength of the linear relationship between mean age at death and each SNP while controlling for the effects of the other variables. Given the evidence for effect modification due to gender within animal models [26] a SNP × gender interaction effect was also tested. Because this interaction effect was not included in base model selection, an associated p-value of ≤0.10 was used to determine inclusion of this interaction effect. For 45 of the 48 SNPs evaluated, this interaction effect was not significant. For these SNPs, positive false discovery rates (pFDR) and *q-*values were computed to control for multiple testing [27]. For the three SNPs where the SNP × gender interaction effect was significant, follow-up gender-specific analysis was conducted. Gender-specific analysis of risk factor frequency was completed. All statistical tests were performed using SAS v9.3 (SAS Institute Inc., Cary, NC).

## Results

### Participant characteristics and environmental risk factors

Of the 3763 participants in 65+RHS, 1192 had genotype information for the 48 SNPs within 15 candidate genes at a sufficient call rate (>90%) for analysis. There are some expected differences between participants included in this analysis and the remaining Iowa 65+RHS cohort. As shown in Table 1, the 1192 participants in this study had a higher mean age at death, smoked less at baseline, had slightly higher baseline BMI, and lower comorbidity at baseline. Participants had to survive to the 6^th^ interview to consent to submit a blood sample, resulting in a higher mean age at death. This was also consistent with less smoking and lower comorbidity in participants whose blood was analyzed, indicating a historically healthier lifestyle. The higher BMI in participants analyzed could be due to a significant difference in the proportion of ever smokers, 28 vs. 35% (p = 0.01).

**Table 1:** Characteristics of 65+ RHS participants by genotyping status.

		Participants Not Genotyped (n = 2481)		Participants Genotyped (n = 1192)	
Age at Death	Mean	86.33		89.46	
	p-value			**p<0.01**	
	Range	66–109		72–107	
	Missing	50		17	
**Characteristic**	**Category**	**N**	**(%)**	**N**	**(%)**
Smoking	No	1790	72.1	892	74.8
	Yes	691	27.9	300	25.2
	p-value				p = 0.08
Drinking Status	Never	886	46.6	487	48.9
	Former	194	10.2	91	9.1
	Low Current	750	39.4	386	38.8
	High Current	72	3.8	32	3.2
	p-value				p = 0.54
	Missing	579		196	
Body Mass Index	Underweight	109	5.4	29	2.7
	Normal	990	48.9	482	44.4
	Overweight	698	34.4	440	40.6
	Obese	230	11.3	134	12.3
	p-value				**p<0.01**
	Missing	454		107	
Comorbidity Count	0	999	40.5	551	46.5
	1	1077	43.7	502	42.4
	>1	389	15.8	131	11.1
	p-value				**p<0.01**
	Missing	16		8	

Comparison of covariables included in the multivariable model between 65+RHS participants with genotype data compared to the rest of the Iowa cohort. These covariables, collected during the baseline interview, were selected because they are aging-related risk factors, and were included in the model to control for confounding. The Iowa 65+RHS cohort is representative of an elderly Iowa, Caucasian population. Expected differences in age (due to blood withdraw at follow-up interview six) and factors historically related to a healthier lifestyle were seen between those genotyped and those not genotyped.

### Association of Minor Allele Homozygotes with Decreased Lifespan

**Figure 1:**
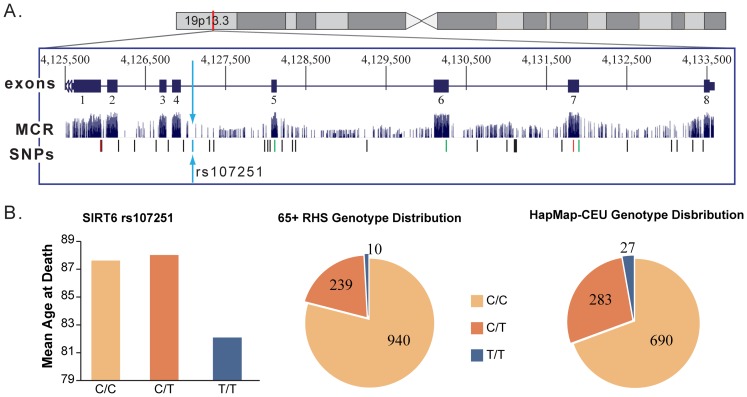
*AKT1* rs3803304 location and adjusted mean age at death and genotype frequency distribution stratified by gender. A. *SIRT6* is located on chromosome 19p13.3, 8491 bp in length with 8 exons [30]. rs107251, indicated by a blue dash and blue arrows, is an intron variant located after exon 4. It is not located in a mammalian conserved region indicating it is not likely a causal variant for lifespan. Several SNPs are located in *SIRT6*; rs107251 is thought to capture all of this information. SNPs denoted by a red dash are missense variants; a green dash denotes a synonymous variant. B. CC genotypes and CT genotypes have a >5-year adjusted mean survival advantage over the TT genotype. Data were calculated by multivariable linear regression with adjustment for BMI, smoking, alcohol consumption and comorbidities. There is a significant genotype distribution difference between the 65+RHS and HapMap-CEU populations (p<0.01).

Homozygous minor allele genotypes within rs2841505 (*SIRT5)*, rs107251 *(SIRT6)*, and rs2536512 *(SOD3)* were associated with a shorter lifespan (Table 2) after controlling for aging-related risk factors (gender, BMI, smoking, alcohol consumption and comorbidity). rs107251 (*SIRT6)* showed the greatest impact on lifespan, the ten cohort members homozygous for the minor T allele had a decreased lifespan compared to those with the CT or the CC genotypes (5.9 and 5.5 years, respectively). As shown in Fig. 1, there was a significant difference in the genotype distributions at this locus between the 65+RHS cohort and the HapMap-CEU population (p<0.01), with an obvious deficit of TT homozygotes in the 65+RHS cohort. The crude association for this SNP was of a similar magnitude; the TT genotype had a mean lifespan of 82.1 years, where CT genotype was 88.0 and CC genotype was also 87.6.

**Table 2:** Genotype frequency, distribution, and genotype-specific mean age at death of the SNPs associated with lifespan.

Gene	Chromosome	rs#	Position	Genotype	Genotype frequency	Age at death	q-value	Partial correlation*
*SIRT3*	11p15.5	rs511744	209089	CC	591	87.2	**0.046**	0.0053
				CT	494	88.0		
				TT	106	88.5		
				Missing	1			
*SIRT5*	6p23	rs2841505	13679574	TT	534	88.1	**0.043**	0.0053
				TG	539	87.3		
				GG	119	86.8		
				Missing				
*SIRT6*	19p13.3	rs107251	4127085	CC	940	87.6	**0.012**	0.0075
				CT	239	88.0		
				TT	10	82.1		
				Missing	3			
*SOD3*	4p15.2	rs2536512	24801315	GG	483	88.9	**0.048**	0.0057
				GA	466	87.8		
				AA	124	87.4		
				Missing	119			
*FOXO3*	6q21	rs4946935	109000742	GG	540	87.3	**0.042**	0.0058
				GA	469	87.7		
				AA	111	88.9		
				Missing	72			

*The partial correlation is a measure of the strength of the relationship between the SNP and mean age at death after controlling for gender, comorbidity count, smoking, alcohol consumption, BMI and the BMI × smoking interaction effect.

Of the 45 SNPs where the SNP × gender interaction effect was not significant, *SIRT3*, *5*, *6*, *FOXO3* and *SOD3* SNPs were associated with age at death. All SNPs gene, rs number, chromosome, chromosome position, genotype frequency, age at death, q-value and partial correlation are given.

Cohort members with the rs2841505 (*SIRT5*) GG genotype had decreased lifespan compared to those with the TG or the TT genotype (0.5 and 1.3 years, respectively). Cohort members with the rs2536512 (*SOD3*) AA genotype had decreased lifespan compared to those with the GA genotype or the GG genotype (0.4 and 1.5 years, respectively).

The remaining SNPs within *SIRT5* and *SOD3* and candidate SNPs within *SIRT1-3*, *GPX1*, *FOXO1*, *SOD2*, and *CAMK4* showed no association between the homozygous minor allele genotype and decreased lifespan (S1 Table).

### Association of Minor Allele Homozygotes with Increased Lifespan

Homozygous minor allele genotypes within rs511744 (*SIRT3)* and rs4946935 (*FOXO3)* were associated with an increased lifespan. Cohort members with the rs511744 (*SIRT3)* TT genotype had an increased lifespan compared to those with the CT or the CC genotype (0.5 and 1.3 years, respectively). Cohort members with the rs4946935 (*FOXO3)* AA genotype had an increased lifespan compared to those with the GA or the GG genotype (1.2 and 1.6 years, respectively).

The remaining SNPs within *SIRT3* and *FOXO3* and candidate SNPs within *SIRT1*, *SIRT2*, *SIRT4*, *FOXO1*, and *TP53* showed no association between the homozygous minor allele genotype and increased lifespan (S1 Table).

### Gender Effect Modification of the Association between Candidate Genetic Variants and Lifespan

**Figure 2:**
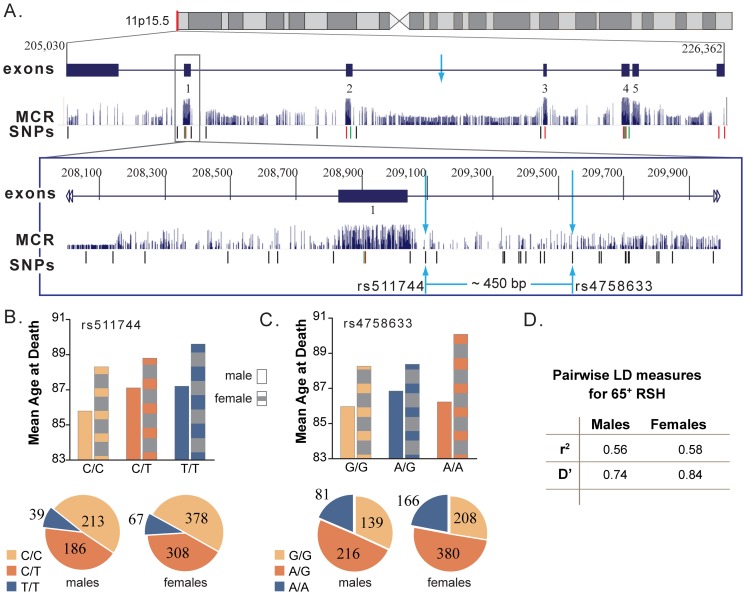
*SIRT3* from chromosomal to base pair level demonstrating the location and characteristics of the two *SIRT3* SNPs significantly associated with adjusted mean age at death. A. *SIRT3* is located on chromosome 11p15.5, 21332 bp in length with 5 exons.[30] Of 12 *SIRT3* SNPs genotyped, two were significantly associated with age at death, rs511744 and rs4758633, denoted by the blue arrows. These two SNPs are approximately 450 base pairs apart, located just after exon 1. Even though very close in physical location, there was a significant gender × rs4758633 interaction effect (p = 0.08) but not rs511744 (p = 0.49). These two SNPs are not in mammalian conserved regions (MCR) indicating they are likely not causal but in LD with the functional variant. SNPs denoted by a red dash are missense variants; a green dash denotes a synonymous variant. B. Adjusted mean age at death for males and females for rs511744. Data were calculated by multivariable linear regression with adjustment for BMI, smoking, alcohol consumption and comorbidities. Genotype frequency distribution did not differ between males and females for rs511744 (p = 0.86) C. Adjusted mean age at death for males and females for rs4758633. Genotype frequency distribution did differ for rs4758633 (p<0.01).D. rs511744 and rs4758633 are in moderate LD (r^2^ value between 0.5-0.8) for both males and females.

**Figure 3:**
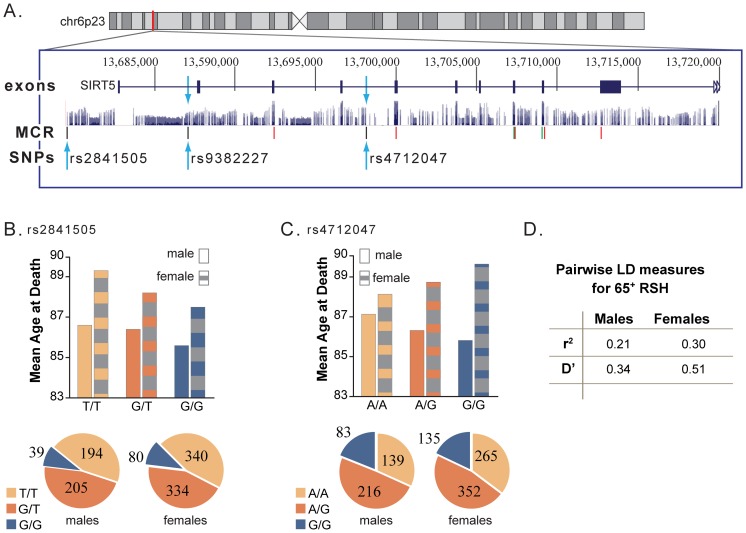
SIRT5 from chromosomal to kilobase level to show the location and characteristics of two SIRT5 SNPs significantly associated with the adjusted mean age at death. A. SIRT5 is located on chromosome 6p23, 37,647 bp long with 8 exons.[30] Blue arrows denote the three SNP genotypes within SIRT5. SNP rs2841505 is an upstream variant in a mammalian conserved region (MCR) and rs4712047 is an intron variant, not in a mammalian conserved region. SNPs denoted by a red dash are missense variants; a green dash denotes a synonymous variant. B. The gender interaction effect with rs2841505 was not significant (p = 0.55). The genotype frequency distribution did not differ between males and females (p = 0.54). C. The gender interaction effect with rs4712047 was significant (p = 0.06). The genotype frequency distribution does not differ between males and females (p = 0.47). D. Pairwise LD estimates suggest rs2841505 and rs4712047 are in low LD.

**Figure 4:**
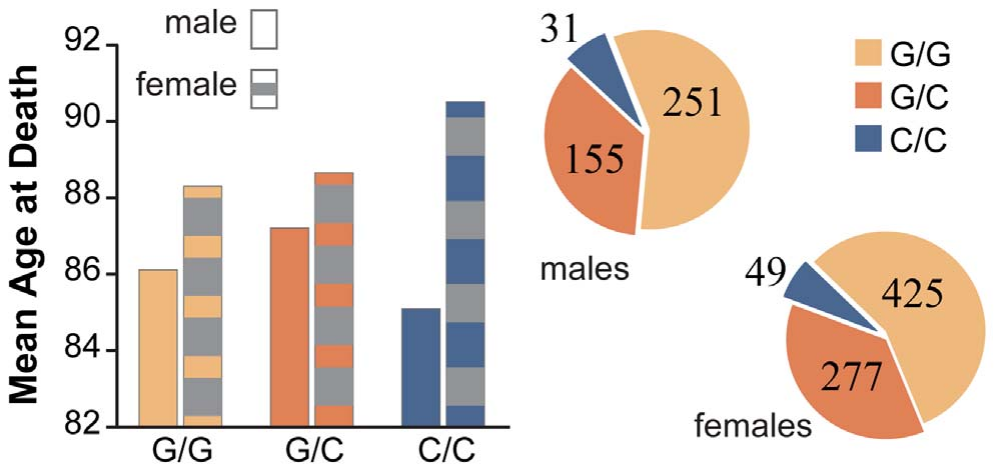
AKT1 rs3803304 location and adjusted mean age at death and genotype frequency distribution stratified by gender. AKT1 is located on chromosome 14q32.33. [30] The gender interaction effect with rs3803304 was significant (p = 0.04). There is no difference in genotype distribution between males and females (p = 0.85).

Gender modified the association between three SNPs (rs4758633 (*SIRT3)*, rs4712047 (*SIRT5*) and rs3803304 (*AKT1*)) and lifespan (p<0.1). In Fig. 2, females with the rs4758633 AA genotype had an increased lifespan compared to females with the GA (0.7 years) or the GG genotype (1.8 years), whereas males with the AA genotype had a decreased lifespan of 0.7 years compared to males with the GA genotype but an increased lifespan of 0.3 years when compared to males with the GG genotype. In Fig. 3, females with the rs4712047 (*SIRT5*) GG genotype had an increased lifespan compared to females with the GA or the AA genotype (0.9 and 1.5 years, respectively), whereas males with GG genotype had a decreased lifespan compared to males with the GA or the AA genotype (0.5 and 1.7 years, respectively). In Fig. 4, females with the rs3803304 (*AKT1)* CC genotype had an increased lifespan compared to females with the GC or the GG genotype (1.8 and 2.2 years, respectively) whereas males with CC genotype had a decreased lifespan compared to males with the GC or the GG genotype (2.1 and 1.0 years, respectively). Risk factor distribution differed between males and females (Fig. 3).

Sensitivity analysis demonstrated the same magnitude and direction of association for all SNPs analyzed.

## Discussion

This study found the association of the *SIRT6* SNP rs107251 minor allele homozygotes (TT) with a decreased lifespan of 5.5 and 5.9 years when compared to the major allele homozygous (CC) and heterozygous (CT) genotypes, respectively, while controlling for BMI, smoking, alcohol consumption and comorbidities. However, it should be noted that only ten cohort members were in the homozygous TT genotype group. Characteristics of this SNP, and the other associated SNPs within this study, suggest they are not causal variants but are in linkage disequilibrium with a functional variant (discussed below). There is accumulating evidence of *SIRT6* impacting lifespan in mammals and now, in humans.

Sirt6-deficent mice develop aging-related abnormalities and signs of premature aging [28]. Male transgenic mice overexpressing Sirt6 were shown to have a 15% increase in median lifespan, where no increased lifespan was observed in female mice [26]. To date, Soerensen *et al*. [2013], is the only other published study which has evaluated the association between *SIRT6* SNPs and lifespan, and found an association with rs107251 *(SIRT6)*. Our results also suggest rs107251 *(SIRT6)* is associated with lifespan thus confirming and extending Sorensen *et al.*'s earlier observation to another population. Additionally, in the current study, adjusted mean age at death was calculated for each genotype allowing for the differences between the genotypes to be analyzed. To our knowledge, this is the first study to include age at death estimates and directly demonstrate the effect of a candidate genotype. Our cohort has a different genotype distribution than the HapMap –CEU population at this locus, with a lower proportion of CT and TT genotypes (Fig. 1). The lower TT frequency could be due to the fact that our population was at minimum 72 years of age; the results of this study suggest people with a TT genotype do not live as long. Lower TT and CT frequency may also be due to underlying population differences between the HapMap-CEU population and the 65+RHS cohort analyzed.

As shown in Fig. 1, *SIRT6* is located on chromosome 19p13.3; it is 8491 base pairs (bp) in length with 8 exons ranging in length from 60 bp to 838 bp [29], [30]. rs107251 is located 1979 bp from the 5′ end, beyond exon 4 and outside a mammalian conserved region [30]. rs107251 is an intron variant, suggesting it is not causal but is in linkage disequilibrium (LD) with a functional variant within *SIRT6* or a neighboring gene. HapMap-CEU analysis of *SIRT6* identified rs107251 as capturing all of the variation within the gene. The SIRT6 protein is a nicotinamide adenine dinucleotide (NAD) dependent enzyme that has been recently shown to interact with other proteins to regulate cellular stress resistance [31]. In male mice, Sirt6 has also been found to decrease phosphorylation in AKT and FOXO3, components of the insulin pathway, contributing to increased lifespan [26]. Interestingly, the decrease in phosphorylation or increased lifespan was not seen in female *Sirt6*-transgenic mice. The region on chromosome 19 where *SIRT6* is located is frequently affected by chromosomal alterations in acute leukemia [29].

Although much more modest than *SIRT6*, this study also found an association of variants in *SIRT3*, *SIRT5*, *FOXO3* and *SOD3* SNPs with age at death (Table 2). The association of human lifespan and *SIRT3* has been more extensively studied compared to *SIRT6*, although with inconsistent results. *SIRT3* was found to be associated with lifespan in two studies [32], [33], a third did not show an association [34], and a fourth had mixed results [35]. *SIRT5* has been associated with an increased brain molecular age [36]. *FOXO3* has also been previously reported to be associated with lifespan [14]. Given the overwhelming body of evidence linking oxidative stress genes to aging these results are not unexpected. However, it is intriguing that studies of the same genes have yielded significant results in some studies but not others. One potential explanation for this could be that many studies only focus on a small number of candidate genes and unmeasured causal variants in weak LD with the genotyped polymorphisms are missed [37]. Additionally, the study design and model selection can substantially influence results. Many lifespan studies employ case-control designs; however, the age used to define “case” and “control” groups differs greatly among studies.

Qualitative interaction effects on final age at death were observed between gender and each of rs4758633 (*SIRT3)*, rs4712047 (*SIRT5)*, and rs3803304 (*AKT1)* (Figs. 2–4, respectively). There were differences in the distribution of risk factors for males and females (Table 3). Gender difference in the association of genotype and lifespan has also been observed for variants in *APOE* [38], [39] and *ADA* [40]. Gender-specific associations have also been found for lung cancer [41], cognitive change [42] and fat distribution [43], [44].

**Table 3:** Characteristics of participants not analyzed *vs*. analyzed, by gender.

		Males Not Analyzed (n = 982)		Males Analyzed (n = 438)		Females Not Analyzed (n = 1499)		Females Analyzed (n = 754)	
Age at Death	Mean	83.8		87.24		88.03		90.77	
	Range	66–109		73–103		67–107		72–107	
	Missing (still alive)	10		2		40		15	
	p-value				**p<0.01** *				**p<0.01** *
**Characteristic**	**Category**	**N**	**(%)**	**N**	**(%)**	**N**	**(%)**	**N**	**(%)**
Smoking	No	399	40.6%	189	43.2%	1391	92.8%	703	93.2%
	Yes	583	59.4%	249	56.8%	108	7.2%	51	6.8%
	p-value				p = 0.38**				p = 0.73**
Drinking Status	Never	185	24.1%	111	29.8%	701	61.8%	376	60.3%
	Former	120	15.6%	51	13.7%	74	6.5%	40	6.4%
	Low Current	419	54.6%	194	52.2%	331	29.2%	192	30.8%
	High Current	44	5.7%	16	4.3%	28	2.5%	16	2.6%
	Missing	214		66		365		130	
	p-value				p = 0.17**				p = 0.91**
Body Mass Index	Underweight	26	3.4%	7	1.8%	83	6.6%	21	3.0%
	Normal	339	43.7%	146	37.0%	651	52.0%	336	48.8%
	Overweight	343	44.2%	197	49.9%	355	28.4%	243	35.3%
	Obese	68	8.8%	45	11.4%	162	12.9%	89	12.9%
	missing	206		43		248		65	
	p-value				**p = 0.03** **				**p<0.01** **
Comorbidity Count	0	408	41.9%	205	47.1%	591	39.6%	346	46.2%
	1	410	42.1%	167	38.4%	667	44.7%	335	44.7%
	>1	156	16.0%	63	14.5%	233	15.6%	68	9.1%
	Missing	8		3		8		5	
	p-value				p = 0.19**				**p<0.01** **
Exercise	Highly Active	340	34.6%	139	31.7%	374	24.9%	157	20.8%
	Moderate	356	36.3%	190	43.4%	573	38.2%	364	48.3%
	Inactive	286	29.1%	109	24.9%	552	36.8%	233	30.9%
	p-value				**p<0.01** **				**p<0.01** **

*Student's t-test p-value compares the mean age at death for participants not analyzed *vs*. analyzed by gender.

**Chi-square p-value compares distribution within categories for participants not analyzed with participants analyzed, by gender.

Differences in life-span between and males and females exist in various regions of the world, with females living a mean of 4.2 years longer than males; this is projected to increase to 4.8 years by the year 2050 [45], [46]. Theories for this difference include females having two X-chromosomes, the suppressing effects of estrogens upon aging-related cell senescence and the favorable cardiovascular effects of hormonal fluctuations in females during their reproductive period [45]. A study in rats showed estrogens were responsible for lower mitochondrial free-radical production observed in females compared to males [47]. Though women in this study are post-menopausal, estrogen may have decreased the oxidative-stress burden throughout life contributing to the increased lifespan. For rs4758633 (*SIRT3)*, the gender-specific genotype frequency distributions were significantly different (p<0.01). Females in the 65+RHS had a higher proportion of the AA genotype than males. This genotype was associated with a greater lifespan in females but not males and could explain a small proportion of the mean difference in lifespan for females and males.

In Figs. 2 and 3, both *SIRT3* and *SIRT5* had one SNP which was significant for males and females combined (rs511744 *SIRT3* and rs2841505 *SIRT5*) but another SNP which showed a significant gender interaction effect (rs4758633 *SIRT3* and rs4712047 *SIRT5*). rs2841505 is a sequence variant located 5′ of *SIRT5*, while the other three SNPs are intron variants. This suggests these four SNPs are not causal but are in LD with a functional variation of the respective, or neighboring, gene. It also suggests estrogen or another factor could be causing the gene or its proteins to act differently in males and females. This may be another contributing factor to inconsistent results among studies (when gender-specific effects are not considered) and highlights the importance of entire genetic coverage and investigation of effect modification due to gender in association studies.

Caution must be taken with these results due to several limitations. First, this study population was a relatively small sample. Only ten cohort members had the homozygous minor allele genotype for rs107251, which demonstrated the greatest impact on lifespan. Second, these analyses were exploratory and a p-value of 0.1 was used to evaluate interaction effects. Third, there may be residual confounding in smoking and other variables. Due to small numbers of smokers only two categories (ever vs. never) were used. Fourth, the minimum age at the time of enrollment into the study in 1982–83 was 65 and blood wasn't collected until the 6^th^ interview during 1987–89. Selective pressures that influenced their mortality, such as caloric restriction of the Great Depression and absence of vaccination, are different than today and may influence results [48]. The effects of caloric restriction may be minimized since BMI was controlled for, although information regarding caloric restriction is not likely captured entirely by BMI. It should be noted that the range of ages in this study might be ideal for studying genetic effects and aging. The GenomeEUtwin project, including more than 20,000 Nordic twins, saw that genetic effects on lifespans were minimal prior to 60 years of age, but genetic effects on lifespan after age 60 were significant and constant to increasing with advancing age [49]. Fifth, these SNPs are not causal but are in LD with functional variants. Further investigation is needed to identify the variation which is impacting age at death. Finally, all exposure information was self-reported from the baseline interview. However, this information has been shown to be relatively accurate [19], [50], [51].

In addition to potentially being an ideal age group this study has other strengths. Death dates were available for an overwhelming majority of this population. Although self-reported, the baseline survey provided information and aging-related risk factors that could be controlled for. In genetic testing, an environmental factor itself is of no direct interest but can be an important component in controlling for potential confounding [52]. Positive false discovery rates were controlled when evaluating the 45 SNPs where the gender × SNP interaction effect was not significant (p>0.1). Utilizing a multivariable linear model allowed for evaluation of the adjusted mean age at death for each genotype.

In conclusion, our data suggest associations between lifespan and several sirtuin and oxidative stress gene SNPs, most notable in *SIRT6*. Qualitative effect modification was significant for variants in *SIRT3*, *SIRT5* and *AKT1*. These findings show the importance of utilizing information provided through animal models in candidate genetic studies. Furthermore, our data suggest that effects of gender and aging-related risk factors should be controlled for in lifespan studies and that stratification by gender may reveal effect modification. This study was exploratory and replication of these findings in other human populations is needed.

## Supporting Information

S1 Table **Genotype frequency, distribution, and genotype-specific mean age at death of the remaining SNPs not associated with lifespan.**
(PDF)

S2 Table **Data of cohort used in this study.**
(XLSX)

## References

[1] Crimmins EM, Finch CE (2012) The genetics of age-related health outcomes. J Gerontol A Biol Sci Med Sci 67:467–469. 22454370 10.1093/gerona/gls101 PMC3326245

[2] Engelfriet PM, Jansen EH, Picavet HS, Dolle ME (2013) Biochemical Markers of Aging for Longitudinal Studies in Humans. Epidemiol Rev 35:132–151. 23382477 10.1093/epirev/mxs011 PMC4707878

[3] Guarente L (2011) Franklin H. Epstein Lecture: Sirtuins, aging, and medicine. N Engl J Med 364:2235–2244. 21651395 10.1056/NEJMra1100831

[4] Tissenbaum HA, Guarente L (2001) Increased dosage of a *sir-2* gene extends lifespan in *Caenorhabditis elegans* . Nature 410:227–230. 11242085 10.1038/35065638

[5] Burnett C, Valentini S, Cabreiro F, Goss M, Somogyvari M, et al (2011) Absence of effects of Sir2 overexpression on lifespan in *C. elegans* and *Drosophila* . Nature 477:482–485. 21938067 10.1038/nature10296 PMC3188402

[6] Merksamer PI, Liu Y, He W, Hirschey MD, Chen D, et al (2013) The sirtuins, oxidative stress and aging: an emerging link. Aging (Albany NY) 5:144–150. 23474711 10.18632/aging.100544 PMC3629286

[7] Lin SJ, Defossez PA, Guarente L (2000) Requirement of NAD and *SIR2* for life-span extension by calorie restriction in *Saccharomyces cerevisiae* . Science 289:2126–2128. 11000115 10.1126/science.289.5487.2126

[8] Houtkooper RH, Pirinen E, Auwerx J (2012) Sirtuins as regulators of metabolism and healthspan. Nat Rev Mol Cell Biol 13:225–238. 22395773 10.1038/nrm3293 PMC4872805

[9] Brunet A, Sweeney LB, Sturgill JF, Chua KF, Greer PL, et al (2004) Stress-dependent regulation of FOXO transcription factors by the SIRT1 deacetylase. Science 303:2011–2015. 14976264 10.1126/science.1094637

[10] Wang F, Nguyen M, Qin FX, Tong Q (2007) SIRT2 deacetylates FOXO3a in response to oxidative stress and caloric restriction. Aging Cell 6:505–514. 17521387 10.1111/j.1474-9726.2007.00304.x

[11] Zhang B, Cui S, Bai X, Zhuo L, Sun X, et al (2013) SIRT3 overexpression antagonizes high glucose accelerated cellular senescence in human diploid fibroblasts *via* the SIRT3-FOXO1 signaling pathway. Age (Dordr) 35:2237–2253. 23494737 10.1007/s11357-013-9520-4 PMC3825003

[12] Luo J, Nikolaev AY, Imai S, Chen D, Su F, et al (2001) Negative control of p53 by *Sir2a* promotes cell survival under stress. Cell 107:137–148. 11672522 10.1016/s0092-8674(01)00524-4

[13] Zeng Y, Cheng L, Chen H, Cao H, Hauser ER, et al (2010) Effects of FOXO genotypes on longevity: a biodemographic analysis. J Gerontol A Biol Sci Med Sci 65:1285–1299. 20884733 10.1093/gerona/glq156 PMC2990269

[14] Willcox BJ, Donlon TA, He Q, Chen R, Grove JS, et al (2008) FOXO3A genotype is strongly associated with human longevity. Proc Natl Acad Sci U S A 105:13987–13992. 18765803 10.1073/pnas.0801030105 PMC2544566

[15] Orsted DD, Bojesen SE, Tybjaerg-Hansen A, Nordestgaard BG (2007) Tumor suppressor p53 Arg72Pro polymorphism and longevity, cancer survival, and risk of cancer in the general population. J Exp Med 204:1295–1301. 17535973 10.1084/jem.20062476 PMC2118619

[16] Malovini A, Illario M, Iaccarino G, Villa F, Ferrario A, et al (2011) Association study on long-living individuals from Southern Italy identifies rs10491334 in the *CAMKIV* gene that regulates survival proteins. Rejuvenation Res 14:283–291. 21612516 10.1089/rej.2010.1114

[17] Newman AB, Murabito JM (2013) The Epidemiology of Longevity and Exceptional Survival. Epidemiol Rev 35:181–197. 23372024 10.1093/epirev/mxs013 PMC4707876

[18] Cornoni-Huntley J, Ostfeld AM, Taylor JO, Wallace RB, Blazer D, et al (1993) Established populations for epidemiologic studies of the elderly: study design and methodology. Aging (Milano) 5:27–37. 8481423 10.1007/BF03324123

[19] Cerhan JR, Torner JC, Lynch CF, Rubenstein LM, Lemke JH, et al (1997) Association of smoking, body mass, and physical activity with risk of prostate cancer in the Iowa 65+ Rural Health Study (United States). Cancer Causes Control 8:229–238. 9134247 10.1023/a:1018428531619

[20] Dutta A, Henley W, Lang I, Llewellyn D, Guralnik J, et al (2011) Predictors of extraordinary survival in the Iowa established populations for epidemiologic study of the elderly: cohort follow-up to "extinction". J Am Geriatr Soc 59:963–971. 21649635 10.1111/j.1532-5415.2011.03451.x PMC3246274

[21] Roerecke M, Rehm J (2012) Alcohol Intake Revisited: Risks and Benefits. Curr Atheroscler Rep 14:556–562. 22864603 10.1007/s11883-012-0277-5

[22] Tremblay A, Bandi V (2003) Impact of body mass index on outcomes following critical care. Chest 123:1202–1207. 12684312 10.1378/chest.123.4.1202

[23] Simonsick EM, Lafferty ME, Phillips CL, Mendes de Leon CF, Kasl SV, et al (1993) Risk due to inactivity in physically capable older adults. Am J Public Health 83:1443–1450. 8214236 10.2105/ajph.83.10.1443 PMC1694862

[24] Fillenbaum GG, Pieper CF, Cohen HJ, Cornoni-Huntley JC, Guralnik JM (2000) Comorbidity of five chronic health conditions in elderly community residents: determinants and impact on mortality. J Gerontol A Biol Sci Med Sci 55:M84–89. 10737690 10.1093/gerona/55.2.m84

[25] Lettre G, Lange C, Hirschhorn JN (2007) Genetic model testing and statistical power in population-based association studies of quantitative traits. Genet Epidemiol 31:358–362. 17352422 10.1002/gepi.20217

[26] Kanfi Y, Naiman S, Amir G, Peshti V, Zinman G, et al (2012) The sirtuin SIRT6 regulates lifespan in male mice. Nature 483:218–221. 22367546 10.1038/nature10815

[27] Storey JD (2002) A direct approach to false discovery rates. Journal of the Royal Statistical Society Series B-Statistical Methodology 64:479–498.

[28] Mostoslavsky R, Chua KF, Lombard DB, Pang WW, Fischer MR, et al (2006) Genomic instability and aging-like phenotype in the absence of mammalian SIRT6. Cell 124:315–329. 16439206 10.1016/j.cell.2005.11.044

[29] Mahlknecht U, Ho AD, Voelter-Mahlknecht S (2006) Chromosomal organization and fluorescence in situ hybridization of the human Sirtuin 6 gene. Int J Oncol 28:447–456. 16391800

[30] Kent W, Sugnet C, Furey T, Roskin K, Pringle T, et al (2002) The human genome browser at UCSC. Genome Res 12:996–1006. 12045153 10.1101/gr.229102 PMC186604

[31] Simeoni F, Tasselli L, Tanaka S, Villanova L, Hayashi M, et al (2013) Proteomic analysis of the SIRT6 interactome: novel links to genome maintenance and cellular stress signaling. Sci Rep 3:3085. 24169447 10.1038/srep03085 PMC3812651

[32] Rose G, Dato S, Altomare K, Bellizzi D, Garasto S, et al (2003) Variability of the *SIRT3* gene, human silent information regulator *Sir2* homologue, and survivorship in the elderly. Exp Gerontol 38:1065–1070. 14580859 10.1016/s0531-5565(03)00209-2

[33] Albani D, Ateri E, Mazzuco S, Ghilardi A, Rodilossi S, et al (2014) Modulation of human longevity by SIRT3 single nucleotide polymorphisms in the prospective study "Treviso Longeva (TRELONG)". Age (Dordr) 36:469–478. 23839864 10.1007/s11357-013-9559-2 PMC3889902

[34] Soerensen M, Dato S, Tan Q, Thinggaard M, Kleindorp R, et al (2013) Evidence from case-control and longitudinal studies supports associations of genetic variation in APOE, CETP, and IL6 with human longevity. Age (Dordr) 35:487–500. 22234866 10.1007/s11357-011-9373-7 PMC3592963

[35] Lescai F, Blanche H, Nebel A, Beekman M, Sahbatou M, et al (2009) Human longevity and 11p15.5: a study in 1321 centenarians. Eur J Hum Genet 17:1515–1519. 19367319 10.1038/ejhg.2009.54 PMC2986679

[36] Glorioso C, Oh S, Douillard GG, Sibille E (2011) Brain molecular aging, promotion of neurological disease and modulation by sirtuin 5 longevity gene polymorphism. Neurobiol Dis 41:279–290. 20887790 10.1016/j.nbd.2010.09.016 PMC3014380

[37] Fallin MD, Matteini A (2009) Genetic epidemiology in aging research. J Gerontol A Biol Sci Med Sci 64:47–60. 19168782 10.1093/gerona/gln021 PMC2691193

[38] Seripa D, Franceschi M, Matera MG, Panza F, Kehoe PG, et al (2006) Sex differences in the association of apolipoprotein E and angiotensin-converting enzyme gene polymorphisms with healthy aging and longevity: a population-based study from Southern Italy. J Gerontol A Biol Sci Med Sci 61:918–923. 16960022 10.1093/gerona/61.9.918

[39] Beydoun MA, Beydoun HA, Kaufman JS, An Y, Resnick SM, et al (2013) Apolipoprotein E e4 allele interacts with sex and cognitive status to influence all-cause and cause-specific mortality in U.S. older adults. J Am Geriatr Soc 61:525–534. 23581910 10.1111/jgs.12156 PMC3628727

[40] Napolioni V, Lucarini N (2010) Gender-specific association of ADA genetic polymorphism with human longevity. Biogerontology 11:457–462. 20174870 10.1007/s10522-010-9266-7

[41] Dresler CM, Fratelli C, Babb J, Everley L, Evans AA, et al (2000) Gender differences in genetic susceptibility for lung cancer. Lung Cancer 30:153–160. 11137199 10.1016/s0169-5002(00)00163-x

[42] Beydoun MA, Ding EL, Beydoun HA, Tanaka T, Ferrucci L, et al (2012) Vitamin D receptor and megalin gene polymorphisms and their associations with longitudinal cognitive change in US adults. Am J Clin Nutr 95:163–178. 22170372 10.3945/ajcn.111.017137 PMC3238459

[43] Heid IM, Jackson AU, Randall JC, Winkler TW, Qi L, et al (2010) Meta-analysis identifies 13 new loci associated with waist-hip ratio and reveals sexual dimorphism in the genetic basis of fat distribution. Nat Genet 42:949–960. 20935629 10.1038/ng.685 PMC3000924

[44] Zillikens MC, Yazdanpanah M, Pardo LM, Rivadeneira F, Aulchenko YS, et al (2008) Sex-specific genetic effects influence variation in body composition. Diabetologia 51:2233–2241. 18839131 10.1007/s00125-008-1163-0

[45] Eskes T, Haanen C (2007) Why do women live longer than men? Eur J Obstet Gynecol Reprod Biol 133:126–133. 17324494 10.1016/j.ejogrb.2007.01.006

[46] Austad SN (2006) Why women live longer than men: sex differences in longevity. Gend Med 3:79–92. 16860268 10.1016/s1550-8579(06)80198-1

[47] Vina J, Borras C, Gambini J, Sastre J, Pallardo FV (2005) Why females live longer than males: control of longevity by sex hormones. Sci Aging Knowledge Environ 2005:pe17. 15944465 10.1126/sageke.2005.23.pe17

[48] Brooks-Wilson AR (2013) Genetics of healthy aging and longevity. Hum Genet 132:1323–1338. 23925498 10.1007/s00439-013-1342-z PMC3898394

[49] Murabito JM, Yuan R, Lunetta KL (2012) The search for longevity and healthy aging genes: insights from epidemiological studies and samples of long-lived individuals. J Gerontol A Biol Sci Med Sci 67:470–479. 22499766 10.1093/gerona/gls089 PMC3326242

[50] Avendano M, Kawachi I, Van Lenthe F, Boshuizen HC, Mackenbach JP, et al (2006) Socioeconomic status and stroke incidence in the US elderly: the role of risk factors in the EPESE study. Stroke 37:1368–1373. 16690902 10.1161/01.STR.0000221702.75002.66

[51] Purser JL, Fillenbaum GG, Pieper CF, Wallace RB (2005) Mild cognitive impairment and 10-year trajectories of disability in the Iowa Established Populations for Epidemiologic Studies of the Elderly cohort. J Am Geriatr Soc 53:1966–1972. 16274380 10.1111/j.1532-5415.2005.53566.x

[52] Yi N (2010) Statistical analysis of genetic interactions. Genet Res (Camb) 92:443–459. 21429274 10.1017/S0016672310000595 PMC3203544

