# Supplementary material for: SIRT6 Minor Allele Genotype Is Associated with >5-Year Decrease in Lifespan in an Aged Cohort
Source: PLoS One. 2014 Dec 26;9(12):e115616. doi: 10.1371/journal.pone.0115616 (PMC4277407; doi:10.1371/journal.pone.0115616)
Supplement: S1 Table — Genotype frequency, distribution, and genotype-specific mean age at death of the remaining SNPs not associated with lifespan. (PDF) [file pone.0115616.s001.pdf]

Table S1. Genotype frequency, distribution, and genotype-specific mean age at death of the remaining SNPs not associated with longevity

Gene	Chromosome	rs#	Position	Genotype	Genotype frequency	Age at death	q-value	Partial correlation*
SIRT3	11p15.5	rs570591	205364	GG	832	87.77	0.186	0.0029
				GA	336	87.41		
				AA	24	85.63		
				Missing	0			
		rs12226697	206056	GG	1098	87.61	0.784	0.0004
				GA	90	87.54		
				AA	3	85.16		
				Missing	1			
		rs3847648	208613	GG	1110	87.60	0.682	0.0007
				GA	79	87.80		
				AA	2	91.24		
				Missing	1			
		rs3782116	213119	AA	760	87.45	0.099	0.0004
				AG	383	87.56		
				GG	48	88.71		
				Missing	1			
		rs1023430	214393	AA	760	87.78	0.105	0.0038
				AG	383	87.59		
				GG	48	85.85		
				Missing	1			
		rs536715	220368	GG	949	87.57	0.799	0.0004
				GA	227	87.81		
				AA	15	86.98		
				Missing	1			
		rs11246020	223067	GG	768	87.62	0.051	0.0051
				GA	382	87.43		
				AA	40	89.92		
				Missing	2			
		rs28365927	226091	GG	833	87.52	0.13	0.0037
				GA	282	86.75		
				AA	20	86.12		
				Missing	57			
		rs227563	226871	CC	629	87.43	0.246	0.0025
				CT	419	87.78		
				TT	80	88.60		
				Missing	64			
		rs939915	227737	TT	688	87.86	0.109	0.0038
				AT	420	87.63		
				AA	63	86.15		
				Missing	21			
SIRT5	6p23	rs9382222	13678265	CC	566	87.53	0.854	0.0003
				CT	484	87.72		
				TT	123	87.46		
				Missing	18			
SOD3	4p15.2	rs8192287	24405666	GG	1063	87.79	0.267	0.022
				GT	127	86.97		
				TT	2	84.06		
				Missing	0			

Gene	Chromosome	rs#	Position	Genotype	Genotype frequency	Age at death	q-value	Partial correlation*
FOXO3	6q21	rs6911407	108973724	CC	401	87.17	0.092	0.004
				CA	593	87.70		
				AA	187	88.89		
				Missing	11			
		rs2802288	109002908	GG	422	87.33	0.151	0.0032
				GA	578	87.56		
				AA	190	88.37		
				Missing	2			
		rs17598747	109048086	AA	932	87.64	0.513	0.0012
				AG	230	87.48		
				GG	17	89.26		
				Missing	13			
		rs9400239	109084356	CC	539	87.30	0.09	0.0042
				CT	511	87.55		
				TT	119	88.68		
				Missing	23			
SIRT1	10q21.3	rs7895833	69293063	AA	896	87.79	0.23	0.0021
				AG	381	87.24		
				GG	59	87.06		
				Missing	0			
		rs12778366	69313085	TT	896	87.70	0.731	0.0005
				TC	272	87.36		
				CC	17	87.62		
				Missing	7			
		rs3758391	69313348	CC	527	88.07	0.072	0.0045
				CT	515	87.20		
				TT	145	87.44		
				Missing	5			
		rs7896005	69321131	GG	500	87.92	0.069	0.0046
				GA	534	87.04		
				AA	156	87.27		
				Missing	2			
		rs2273773	69336604	TT	1032	87.60	0.692	0.0006
				TC	151	87.91		
				CC	6	86.06		
				Missing	3			
		rs1467568	69345164	GG	501	88.10	0.068	0.0046
				GA	532	87.21		
				AA	156	87.44		
				Missing	3			
		rs2234975	69348084	CC	978	87.54	0.283	0.0022
				CT	193	88.17		
				TT	16	89.05		
				Missing	5			
SIRT4	12q24.31	rs2522138	119226527	AA	829	87.31	0.097	0.0024
				AG	341	87.97		
				GG	0			
				Missing	22			

Gene	Chromosome	rs#	Position	Genotype	Genotype frequency	Age at death	q-value	Partial correlation*
SIRT2	19q13.2	rs2015	44061209	AA	410	87.59	0.119	0.0036
				AC	561	87.32		
				CC	212	88.35		
				Missing	9			
		rs9941448	44070644	CC	1035	87.63	0.761	0.0005
				CT	147	87.61		
				TT	10	86.18		
				Missing	0			
		rs10410544	44077372	CC	349	87.39	0.648	0.0007
				CT	578	87.77		
				TT	154	87.70		
				Missing	11			
		rs4802998	44079556	AA	439	87.46	0.588	0.0009
				AG	568	87.79		
				GG	184	87.34		
				Missing	1			
		rs10405150	44079759	TT	1035	87.5	0.341	0.0018
				TC	146	88.25		
				CC	6	88.92		
				Missing	5			
TP53	17p13.1	rs1042522	7520197	GG	384	87.70	0.175	0.003
				GC	434	87.36		
				CC	72	88.79		
				Missing	2			
GPX1	3p21.31	rs1800668	49370761	CC	575	87.89	0.39	0.016
				CT	480	87.39		
				TT	106	87.50		
				Missing	31			
SOD2	6q25.3	rs4880	160033862	TT	308	87.94	0.154	0.0033
				TC	550	87.73		
				CC	300	87.03		
				Missing	34			
		rs2755209	40035804	AA	439	87.38	0.467	0.0013
				AC	551	87.63		
				CC	189	88.04		
				Missing	13			
		rs2701858	40036389	CC	1030	87.62	0.219	0.0026
				CT	148	87.60		
				TT	9	84.03		
				Missing	5			
		rs2721068	40037712	TT	664	87.58	0.938	0.0001
				TC	447	87.65		
				CC	98	87.82		
				Missing	3			
FOXO1	13q14.11	rs17446614	40037877	GG	860	87.62	0.084	0.0042
				GA	295	87.47		
				AA	36	89.88		
				Missing	1			

Gene	Chromosome	rs#	Position	Genotype	Genotype frequency	Age at death	q-value	Partial correlation*
FOXO1	13q14.11	rs7317254	40115849	AA	554	87.52	0.669	0.0007
				AG	501	87.59		
				GG	122	88.07		
				Missing	15			
		rs1334241	40121110	GG	762	87.59	0.099	0.004
				GA	374	87.58		
				AA	54	89.44		
				Missing	2			
		rs2297627	40131931	TT	548	87.53	0.559	0.001
				TC	485	87.56		
				CC	126	88.18		
				Missing	33			
CAMK4	5q22.1	rs10491334	110800303	CC	752	87.56	0.551	0.001
				CT	366	87.36		
				TT	54	86.66		
				Missing	20			
